# Inkjet and Related Printing Technologies for Plant Systems: From Physiological Monitoring to Functional Biofabrication

**DOI:** 10.3390/biomimetics11100763

**Published:** 2026-10-21

**Authors:** Xiukun Wang, Xing Zhang, Guanghui Wang, Bokai Feng, Yang Zhang

**Affiliations:** 1 School of Mechanical Engineering, Shenyang Ligong University, Shenyang 110159, China; 2 Institute of Applied Ecology, Chinese Academy of Sciences, Shenyang 110016, China; 3 School of Equipment Engineering, Shenyang Ligong University, Shenyang 110159, China; wgh860625@163.com; 4 Department of Mechanical Engineering, Tsinghua University, Beijing 100084, China; bkfeng@mail.tsinghua.edu.cn; 5 Mechanical Industry Key Laboratory of Intelligent Robotics Technology for 3C Product, The Sino-German College of Intelligent Manufacturing, Shenzhen Technology University, Shenzhen 518118, China

**Keywords:** inkjet printing, plant bioelectronics, physiological monitoring, functional biofabrication, green bioprinting

## Abstract

Featuring non-contact, high-precision, and multi-material deposition, inkjet and related printing technologies offer a new alternative technique for plant systems. However, the spatial heterogeneity, continuous growth, and sensitivity of plant tissues to external perturbations make it difficult for conventional methods to target specific sites on living organs. This review evaluates thermal and piezoelectric inkjet printing, together with aerosol jet and electrohydrodynamic printing for plant applications. Moreover, it relates the jetting behavior of the printhead to interfacial processes between droplets and plant surfaces. Practical performance is assessed across four dimensions: effective feature size, organ-scale throughput, potential biological damage, and the physiological compatibility of printing inks with plants. Applications are then organized within a framework comprising five tiers: monitoring, delivery, sensing, biofabrication, and intelligent systems. Specifically, recent studies have demonstrated that these printing approaches can be employed for surface labeling and growth tracking of plant organs, localized delivery of agricultural inputs, fabrication of flexible sensors, and construction of living materials (e.g., plant cells). Combined with artificial intelligence technology, these capabilities could form a closed-loop system spanning sensing to feedback, with printing as the precision actuator, potentially shifting plant management from passive observation to active regulation. Finally, this review discusses the challenges of living-interface dynamics, ink biocompatibility, multiscale integration, and data intelligence. It further outlines the prospects of these technologies for programmable plants and living agricultural interfaces. By bridging printing technologies, plant systems, and functional biofabrication, this review provides a unified framework to guide the development of intelligent and sustainable agricultural systems.

## 1. Introduction

From model species such as Arabidopsis thaliana to major crops including rice and tomato, plant systems have long served as central subjects in the life sciences and agricultural research. Unlike conventional engineering substrates such as glass, silicon wafers, or polymer films, plants are not uniform, static supporting surfaces but living systems characterized by spatial heterogeneity, continuous growth, and pronounced sensitivity to perturbation [1]. Different organs of the same plant, and even different regions of a single leaf, can differ significantly in water content, metabolic state, and stress response [2]. Conventional whole-plant spraying, immersion treatment, and coarse-scale dosing struggle to act precisely on specific tissues, cell populations, or localized regions [3], whereas mechanical puncture, microinjection, electrode implantation, and large-area chemical treatment may induce damage, wound stress, or defense responses that alter the very physiological state under investigation [4]. Moreover, stomatal opening and closure, ion fluxes, metabolite fluctuations, and volatile emission exhibit distinct temporal dynamics that destructive sampling or offline detection cannot record continuously and faithfully [5]. Plant science and precision agriculture therefore require an operational modality that simultaneously offers high spatial resolution, low perturbation, local addressability, and real-time feedback. Moreover, from a biomimetic perspective, plants can be viewed as dynamic living interfaces. The hierarchical surfaces, continuous growth, transport pathways, and adaptive responses may inform the design of printing processes and functional devices.

Inkjet and related printing offer a viable route to meet this need. Its non-contact deposition applies materials directly onto the plant surface with negligible physical damage, making it well suited to fragile tissues that readily trigger defense responses upon mechanical contact [6,7]. Inkjet-based bioprinting can achieve controlled deposition of bioactive substances and living cells at scales approaching a single cell or droplet, while preserving cell viability and function by minimizing the mechanical stress imposed during printing [8]. In addition, the multi-material deposition capability of inkjet printing and its bioprinting derivatives allows dyes, markers, growth regulators, conductive materials, and even living cells to be printed sequentially or in combination [9,10], providing a technical basis for integrating labeling, delivery, and sensing within a single process chain. Laser-induced forward transfer (LIFT) is another non-contact method for the localized deposition of cells and biomaterials. It uses a pulsed laser to transfer material from an absorbing layer on a donor substrate, providing high spatial precision without direct mechanical contact. However, its application requires specialized equipment and suitable donor materials, while laser-induced heating may affect sensitive biological samples [11].

More importantly, these methods are digital, on-demand deposition techniques in which placement, dose, and timing are all programmable [12], so that targeted intervention schemes can, in principle, be generated from phenotypic maps or real-time imaging. At the same time, the cost and throughput advantages of thermal and piezoelectric inkjet systems [13] lend engineering feasibility to their extension into greenhouse and even field settings. These printing technologies can therefore be seen as more than a microscale fabrication tool but as a potential operational platform linking precision manufacturing, plant physiology, and data intelligence. Although relevant studies have begun to emerge, existing reviews have not yet integrated this interdisciplinary field adequately. Most published work has focused on 3D bioprinting of plant cells and tissues [14,15] and on bioinks tailored for plant bioprinting [16]. Such studies provide an important basis for classifying materials and processes, but they seldom address in situ operation on living plants, closed-loop feedback, or the progressive relationships among applications. In particular, a clear and scalable analytical framework is still lacking for how inkjet and related printing evolves from simple labeling into a plant-manipulation platform that combines monitoring, delivery, sensing, biofabrication, and intelligent decision-making.

**Figure 1:**
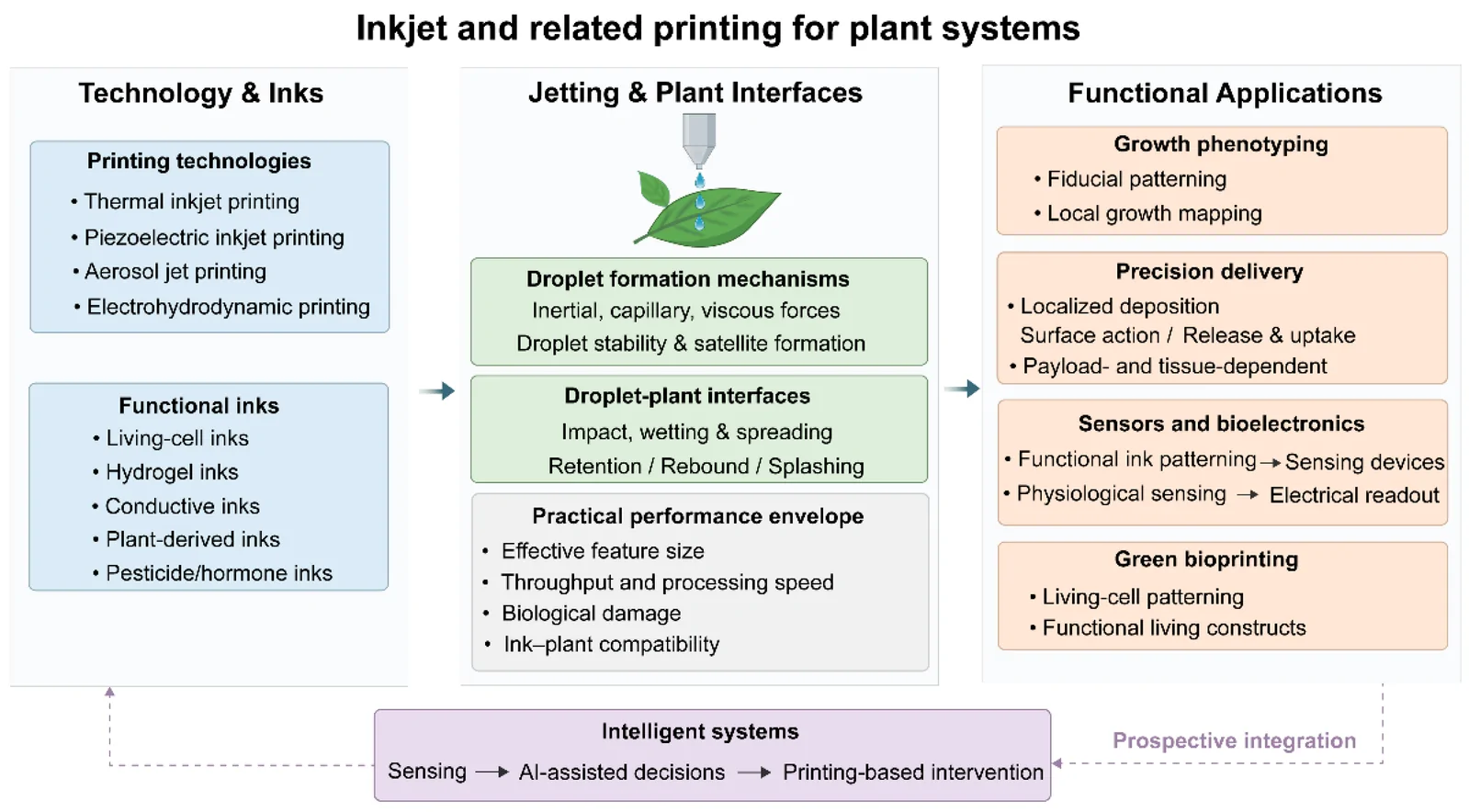
Schematic overview of inkjet and related printing technologies for plant applications.

Accordingly, this review proposes a five-tier integrative framework comprising monitoring, delivery, sensing, biofabrication, and intelligent systems (Figure 1). We first introduce the printing technologies and functional inks applicable to plant systems, including thermal and piezoelectric inkjet printing, aerosol jet printing, and electrohydrodynamic printing. We then evaluate their practical performance in terms of effective feature size, throughput at the organ scale, potential biological damage, and the physiological compatibility of printing inks with plants. The interfacial processes that occur after droplets reach plant surfaces are subsequently analyzed to establish a mechanistic basis for the functional applications. On this basis, we review applications in plant phenotypic labeling, growth tracking, precision delivery, plant bioelectronics, and functional biofabrication. Evidence from non-plant systems is included only when it provides transferable insights into printing mechanisms or engineering constraints, rather than direct evidence of plant compatibility or physiological performance. Finally, we discuss the integration of these printing technologies with artificial intelligence, together with the remaining challenges and future prospects. Through this framework, we aim to show that the value of inkjet and related printing lies not only in material deposition, but also in providing plant systems with a low-perturbation, spatially addressable, and potentially feedback-enabled mode of engineered intervention.

## 2. Inkjet and Related Printing Technologies for Plant Systems

**Figure 2:**
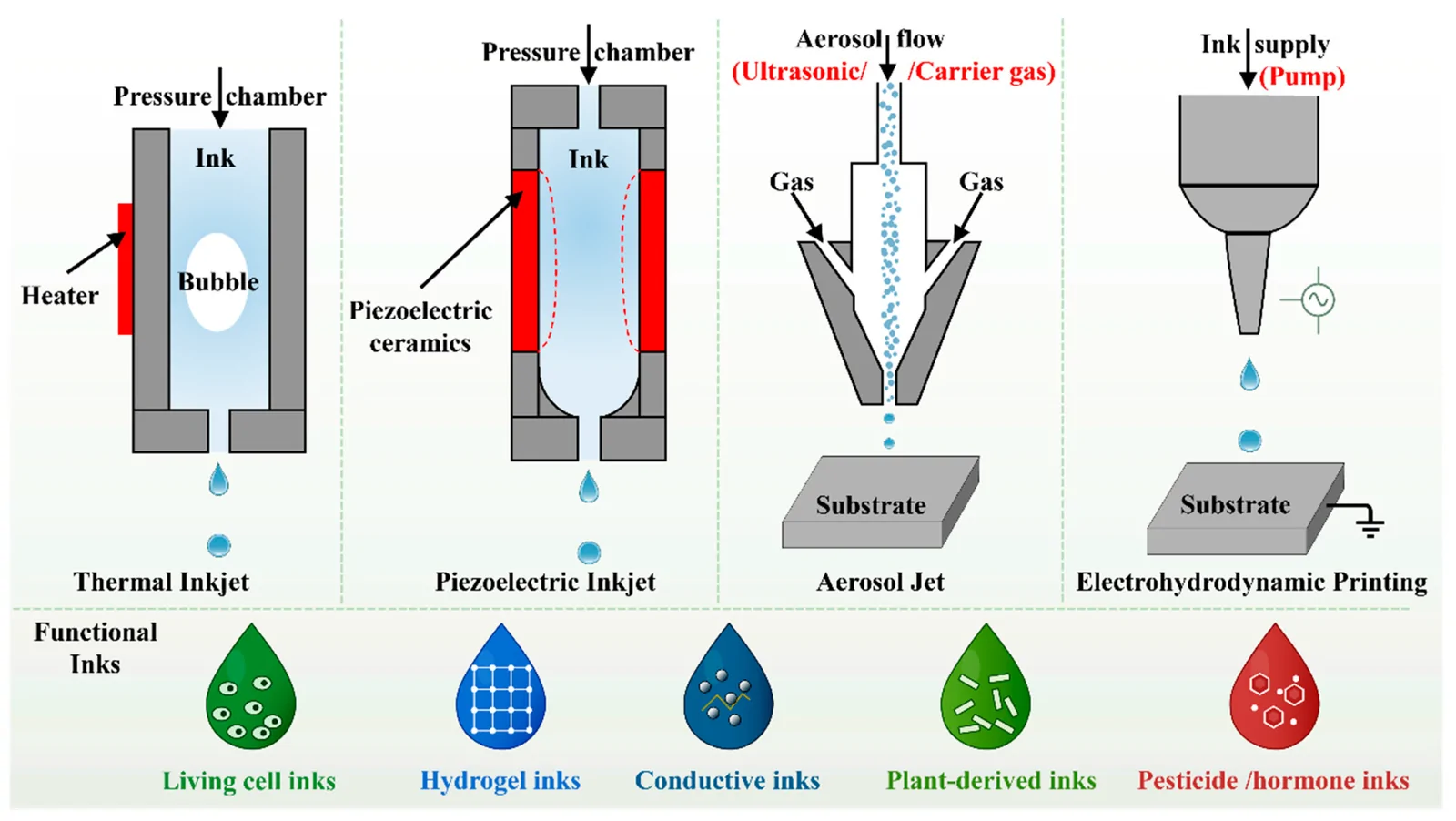
Inkjet and related printing technologies and functional inks for plant systems.

Four printing technologies are directly relevant to plant systems: thermal inkjet printing, piezoelectric inkjet printing, aerosol jet printing, and EHD printing (Figure 2). They differ mainly in printable ink viscosity, droplet/feature size, the thermal or electrical load on living tissue, and hardware maturity and cost. Table 1 compares representative studies along these dimensions, and the following Section 2.1, Section 2.2, Section 2.3 and Section 2.4 discuss each technology in turn, emphasizing its working principle and its specific opportunities and limitations for plant use.

### 2.1. Thermal Inkjet Printing

Thermal inkjet printing is a well-established drop-on-demand (DOD) method. A microscale resistive heater vaporizes a small volume of the ink within a few microseconds, and the resulting vapor bubble expands to eject a droplet through the nozzle [31]; the transient temperature at the heating element can exceed 300 °C [32]. The technology is highly commercialized and is already widely used in office and industrial settings. A typical thermal inkjet system generates droplets of roughly 2–180 pL at heating-pulse frequencies of about 1–5 kHz (pulse widths of ~3–6 μs) [31], with droplet size governed primarily by the thermal gradient, ink viscosity, and pulse frequency. Because these parameters are independently controllable and reproducible, the method affords high-precision patterned deposition.

For bioprinting, local heating accompanies droplet ejection, but the droplet and any cells it carries are exposed to elevated temperature only briefly, and the overall temperature rise is typically limited to ~4–10 °C. Mammalian-cell studies have reported post-printing viabilities of approximately 90% under optimized conditions [17], indicating that transient thermal exposure can be compatible with living material. These findings are cited here mainly to illustrate the process-level effect of thermal jetting rather than to imply direct transferability to plant cells. Thermal inkjet accommodates low-viscosity aqueous solutions, biopolymers, and cell suspensions, and it has been applied to build in vitro tissue models in mammalian systems. In plant research, thermal inkjet is better suited to controlled microdroplet deposition, gentle patterning of reagents and cells, and low-cost fabrication of flexible sensors and microneedle coatings than to whole-cell printing. It therefore mainly serves the delivery and sensing tiers of the framework above.

### 2.2. Piezoelectric Inkjet Printing

Piezoelectric drop-on-demand (DOD) printing relies on a voltage pulse to deform a piezoelectric element, launching a pressure wave within the fluid channel that ejects a single droplet at the nozzle orifice [33]. Droplet formation depends mainly on the balance among viscosity, surface tension, and inertia, so the ink’s rheology often matters more to print quality than the printhead does [34]. Unlike thermal inkjet, this approach requires no transient local heating and is therefore gentler toward thermally sensitive and bioactive materials, which underpins its suitability for handling cells and active molecules. In practice it spans a far wider viscosity range than thermal inkjet (from a few mPa·s up to ~10^5^ mPa·s with dedicated dispensers), giving picoliter-to-nanoliter droplets at ~0.5–1 kHz at moderate cost and maturity (Table 1).

Biocompatibility studies in mammalian cells have reported immediate viabilities of approximately 82–96% after piezoelectric jetting [20], while mechanistic studies indicate that transient pressure, shear stress, and membrane deformation during nozzle passage and ejection are important determinants of cell damage [35,36]. These studies are used here to identify general jetting-related mechanical stresses rather than to infer plant-cell responses directly. Plant cells differ substantially because their rigid cell walls may provide additional mechanical protection, whereas their larger size, vacuoles, aggregation tendency, and the osmotic fragility of protoplasts introduce distinct vulnerabilities. Therefore, direct experimental validation in plant cells remains necessary.

Taken together, piezoelectric inkjet combines three advantages: non-thermal actuation, controllable low-volume dispensing, and good cell compatibility. These advantages could make piezoelectric inkjet promising for plant systems, such as the handling of protoplasts. Nevertheless, direct experimental validation remains necessary. Its stability depends on how nozzle geometry, particle or cell state, driving waveform, and ink rheology act together, so each new system still has to be optimized individually.

### 2.3. Aerosol Jet Printing (AJP)

AJP is a non-contact, direct-write additive method that is particularly well suited to fabricating functional patterns on irregular, flexible, or curved substrates. The process comprises two steps: the functional ink is first atomized, either pneumatically or ultrasonically, into micrometer-scale aerosol droplets, which are then carried by a gas stream and aerodynamically focused by an annular sheath gas to form a narrow, stable deposition beam [37]. Because a defined working distance is maintained between the nozzle and the substrate without physical contact, AJP can directly write high-resolution patterns on both rigid and flexible substrates, such as polyimide, paper, glass, and silicon, while imposing minimal perturbation on ultrathin or fragile surfaces [37,38]. In the cases compiled in Table 1, aerosol-jet line widths range from ~7 to ~100 μm; the trade-off is relatively complex, with higher-cost atomization hardware, which now limits low-cost field deployment.

Material versatility is a further advantage of AJP. Its tolerance to ink viscosity is markedly broader than that of conventional inkjet, allowing metal nanoparticles, conductive polymers, carbon-based materials such as graphene and carbon nanotubes, and biocompatible polymers to be printed [38]. Combined with its maskless, multilayer, multi-material direct-write capability, AJP can stack conductive, dielectric, and sensing layers on a single substrate without photolithography, aligning naturally with the demands of plant-health-monitoring devices for flexibility, heterogeneous integration, and low cost.

This potential has been demonstrated in living-plant sensing. Baraniecki et al. fabricated conductive microneedle electrodes using a fully printed process that combined aerosol jet and inkjet printing and integrated passive near-field communication (NFC) readout to achieve minimally invasive, real-time, wireless impedance monitoring of total ionic conductivity in plant leaves, validated across several plant species with differing leaf anatomies [39]. Building on this approach, coupling microneedles with recognition units such as ion-selective membranes, enzymes, or aptamers, AJP may further support in-field, in situ detection of metabolites, hormones, and even pathogen markers [9,40].

### 2.4. Electrohydrodynamic (EHD) Printing

EHD printing uses an electric field to draw a conductive liquid directly, forming a Taylor cone at the nozzle orifice from which a fine jet or on-demand droplets are pulled. Because the droplets emanate from the cone apex, they can be considerably smaller than the nozzle’s inner diameter, giving the method a high resolution, a controllable deposition, and a relatively broad material tolerance [41]. By adjusting the voltage waveform and feed rate, droplet volume and deposition position can be tuned on demand at the micro- and nanoscale [42]; further optimization of the auxiliary-electrode geometry and process parameters improves the uniformity of the electric field, yielding fine microarrays with consistent line width across a range of substrates [43]. Such resolution, however, requires kV-level fields near the substrate that may harm living tissue, while low single-nozzle throughput and strict process control still limit field use (Table 1).

Material versatility is a notable strength of EHD printing. It can process metallic inks such as nanosilver to produce highly conductive patterns [41], and its tolerance to high-viscosity systems also allows the printing of polymers, such as polycaprolactone, as well as nanocomposite inks [44,45]. At the system and process levels, multi-nozzle cooperative printing alleviates the throughput bottleneck of a single nozzle while suppressing inter-nozzle electric-field crosstalk [46], and data-driven approaches such as machine learning have been used to predict printing behavior and optimize difficult process parameters, further improving stability and operability [47].

Direct experimental validation of EHD printing in living plant systems remains limited. Therefore, the studies summarized above are included primarily to define its process capabilities and engineering constraints. On this basis, EHD printing may have two potential roles in plant research. The first is the fabrication of conductive microcircuits and flexible sensing interfaces for monitoring water status, nutrients, or pathogen-related signals [41]. The second is the construction of biomimetic microfibers or three-dimensional scaffolds through EHD variants such as melt electrowriting, providing a controllable platform for studying the alignment, adhesion, and differentiation of plant cells within defined topographies [44,45]. EHD printing can therefore serve as an important bridge between high-resolution fabrication and the study of plant interfaces.

### 2.5. Functional Inks Tailored for Plant Systems

To accommodate the structural and physiological characteristics of plant systems, functional inks must shift from general-purpose formulations toward application-oriented design. The most representative inks reported to date can be grouped into five functional categories: living-cell inks, hydrogel inks, conductive inks, plant-derived inks, and pesticide/hormone inks, corresponding respectively to the needs of carrying living matter, constructing a microenvironment, conferring electrical functionality, emphasizing sustainability, and achieving precise delivery.

Living-cell inks form the basis of plant biofabrication. The central challenge is to reduce shear-induced damage through a protective matrix while maintaining the viability and metabolic activity of plant cells or microalgae. Polysaccharide hydrogels such as alginate are shear-thinning: their viscosity decreases under flow and recovers rapidly after deposition, thereby striking a balance between printability and cell protection [14]. In principle, such inks could enable living “cell factories” for the continuous production of secondary metabolites, although to date this has been demonstrated mainly in microalgal or suspension systems rather than in printed whole-plant constructs.

Hydrogel inks primarily provide a biomimetic microenvironment for cells after printing. An ideal formulation must simultaneously balance rheological performance, shape fidelity, and biocompatibility. Alginate/methylcellulose composites can markedly improve structural fidelity [48], and in situ ionic crosslinking through a coaxial nozzle further enables curing during printing, shaping the construct while limiting the perturbation to cells caused by post-processing [49]. Future efforts should focus on developing dynamic hydrogels that respond to the evolving needs of growing cells and emulate the mechanical characteristics of the plant cell wall.

Conductive inks link plant printing with bioelectronics. Incorporating conductive components such as carbon nanotubes, graphene, or PEDOT:PSS endows hydrogels or flexible substrates with electrical functionality [50], enabling the direct writing of plant electrodes, sensors, and interconnects, and, in turn, the monitoring of physiological processes such as electrical signaling and water transport. These rigid conductive fillers serve device and interface fabrication rather than acting as a cell-culture matrix, and so they are distinct in role from the wall-mimicking hydrogels above. Recent studies on flexible, wearable sensing for living plants offer a systematic reference for translating such conductive interfaces into plant-health monitoring.

Plant-derived inks emphasize material sustainability. Pea protein, soy protein [51], cellulose nanofibers, and algal polysaccharides such as carrageenan can all serve as matrices; they are widely available and biocompatible, and some exhibit intrinsic gelling ability. The direct printing of seafood analogues from a co-extract of red-algae protein and polysaccharides further illustrates the potential of such sustainable feedstocks in food manufacturing [52]. Although drawn from food manufacturing rather than plant application, it substantiates their processing potential.

Pesticide/hormone inks target precision agriculture, with the core idea of encapsulating chemical agents or plant hormones within degradable or controlled-release matrices so that printing can achieve site-specific, dose-controlled delivery. Prior work has embedded nanoparticle-loaded micronutrients in a degradable matrix and tailored the release profile to different plant growth stages [53]; degradable and recyclable 3D-printed pheromone/agent carriers have likewise been validated for field pest control [54]. This strategy can serve both to study localized hormonal responses and as a technological prototype for future printed fertilization, agrochemical application, and single-plant intervention.

Across these ink categories, additives offer a means of tuning printability beyond the choice of functional material. Poloxamers can help control surface tension and particle dispersion, while glycerol and propylene glycol modify flow properties and help reduce printhead blockage [55]. Natural dispersants offer further options; for example, gum arabic has been used in aqueous carbon-nanotube inks suitable for inkjet printing [56]. However, improved dispersion must be balanced against the effects of residual additives on the printed material and any additional processing needed to remove them [57]. For plant applications, these strategies should be evaluated against jetting stability, deposition on the target organ, and the intended biological or electrical function, with plant compatibility verified for each formulation.

### 2.6. Practical Performance Envelope of Inkjet and Related Printing Technologies for Plant Applications

During inkjet printing, the jetting of functional inks involves complex free-surface evolution and deformation. Droplet formation and shaping are generally governed by the coupled effects of inertial, capillary, and viscous forces [58,59,60]. These effects are commonly characterized using several dimensionless parameters. The Reynolds number (*Re*) represents the ratio of inertial to viscous forces, while the Weber number (*We*) describes the relative importance of inertial and capillary forces. The Ohnesorge number (*Oh*) integrates the relative contributions of viscous, inertial, and capillary effects:

*Re* = *ρvd*/*μ* (1)

*We* = *ρv*^2^*d*/*σ* (2)

(3) Oh=μ/ρσd=We/Re

*Z* = 1/*Oh* (4)

where *ρ* is the ink density, *v* is the characteristic jetting velocity, *d* is the characteristic length, typically the nozzle diameter, *μ* is the dynamic viscosity, and *σ* is the surface tension. Collectively, these parameters provide a theoretical framework for understanding and predicting stable droplet formation, satellite droplet generation, and nozzle failure. *Re*, *We*, and *Oh* provide useful first-order descriptors for conventional droplet jetting, particularly drop-on-demand inkjet printing. They do not define a universal operating window for AJP or EHD printing, for which aerosol-transport or electric-field-related parameters must also be considered, as discussed in Section 2.3 and Section 2.4.

However, for plant-oriented formulations, printability criteria based on *Re*, *We*, and *Oh* must be considered alongside plant-specific physiological constraints. These include biocompatibility, suitable wettability of the plant cuticle, and the avoidance of phytotoxic solvents or surfactants [61,62,63]. Collectively, these coupled hydrodynamic and physiological constraints define the practical performance envelope of inkjet printing for plant applications, as summarized in Table 2. Within this envelope, the practical utility of inkjet printing depends on four interrelated performance dimensions: the effective feature size that can be reproducibly deposited on living organs, organ-scale printing throughput, the mechanisms that may cause cellular or tissue damage during jetting and deposition, and the physiological compatibility of the ink with the plant. These four dimensions are discussed below.

#### 2.6.1. Effective Resolution and Feature Size

The nominal resolution of a printhead is commonly expressed in terms of dots per inch (dpi), nozzle diameter, or minimum line width. However, these specifications cannot be directly equated with the effective resolution achieved on plant surfaces. Previous studies have produced deposition patterns with feature sizes ranging from tens to hundreds of micrometers on leaves, stems, and seedlings [6,7]. From the mechanistic perspective, the final feature size is jointly determined by the initial droplet size and the extent of spreading after deposition. In addition, placement accuracy is also influenced by the jetting direction, the nozzle-to-organ distance, and plant motion. Consequently, the minimum line width reported on engineered substrates should not be directly interpreted as the effective resolution on living plant organs. The relevant interfacial mechanisms are discussed at the beginning of Section 3.

#### 2.6.2. Throughput and Processing Speed

Droplet jetting frequency describes the ability of the printhead to generate droplets per unit time. However, it does not directly represent processing throughput at the organ scale [33,46]. The actual processing speed also depends on the number of nozzles, deposition dose, printing area, pattern complexity, number of scanning passes, and motion path [46,65,66,67]. Therefore, organ-scale throughput is jointly determined by droplet generation capacity and effective deposition efficiency. The latter is constrained by the requirements for positioning, scanning, and repeated deposition. Therefore, the practical efficiency of different printing technologies should be evaluated according to the time required to process a target organ or complete a specified pattern, rather than by comparing jetting frequencies alone.

#### 2.6.3. Mechanisms of Cellular and Tissue Damage

The printing process may affect both the living payloads carried by the ink and the plant tissues subjected to printing. Suspended cells or protoplasts may experience transient heating, pressure, shear stress, membrane deformation, and osmotic stress [17,35,36]. Plant organs may also be affected by droplet impact, solvents, surfactants, or externally applied electric fields [41,61,62]. The extent of such damage depends on the magnitude and duration of local stresses, rather than on the type of printing technology alone. Therefore, damage assessment should consider both post-printing cell viability and the physiological function of the host plant.

#### 2.6.4. Ink–Plant Compatibility

The applicable range of plant-oriented inks is defined by the overlap between the ink printability window and the physiological tolerance window of the plant [34,64]. This compatibility depends on ink composition, deposited dose, exposure duration, plant species, and organ type [62,63]. The rheological and interfacial properties of the ink may vary with its formulation and environmental conditions, thereby affecting jetting stability [33,34]. In parallel, plant tolerance may vary with species, organ type, physiological state, deposited dose, and exposure duration [62,63]. Therefore, the overlap between these two windows should be regarded as dynamic and condition-dependent rather than as an intrinsic material property. Accordingly, current findings are generally applicable only to specific ink formulations and experimental conditions.

In summary, the practical performance of inkjet and related printing technologies in plant systems is defined by four dimensions: effective feature size, organ-scale throughput, biological damage, and ink–plant compatibility. These dimensions reflect droplet formation and deposition, processing efficiency at the organ scale, biophysical perturbations associated with the printing process, and the physiological compatibility between the ink and the plant, respectively. Given the interdependence of these dimensions, the performance ranges reported in the literature should be regarded as operating windows defined by specific printing technologies, ink formulations, plant systems, and experimental conditions, rather than as universal performance limits. This performance framework provides a basis for comparing the suitability of different printing technologies across plant applications in the following section.

## 3. Functional Interfaces Between Inkjet Printing and Plant Systems

Beyond the printhead-level performance envelope discussed in Section 2.6, the functional outcome of plant printing also depends on the interfacial processes occurring after droplets reach the plant surface. For the droplet-based jetting methods described above, droplets may be retained, rebound, spread, or break up upon impact [68]. In addition to the relative contributions of inertial, capillary, and viscous forces described by the Weber and Reynolds numbers, deposition behavior is influenced by leaf wettability, epicuticular waxes, microscale roughness, trichomes, and local curvature [69,70]. Therefore, deposition performance measured on uniform engineered substrates, such as glass and polymers, cannot be directly extrapolated to living plant organs. Once a droplet is retained on the surface, its subsequent spreading and drying are governed by the dynamic contact angle, solvent evaporation, and internal fluid flow. Contact angle hysteresis can be expressed as:

Δ*θ* = *θ_A_* − *θ_R_* (5)

where *θ_A_* and *θ_R_* are the advancing and receding contact angles, respectively. The larger contact angle hysteresis generally restricts droplet motion and may promote solute accumulation near the droplet edge during evaporation [71]. Meanwhile, Marangoni flow and evaporation-induced capillary flow regulate mass transport within the droplet. These internal flows can consequently affect the uniformity and boundary morphology of the deposited pattern [72,73].

Moreover, the required deposition state varies across applications. Plant marking requires sufficient droplet retention and controlled spreading, whereas excessive spreading can reduce spatial positioning accuracy [74]. For localized delivery, the outcome is further influenced by cuticular penetration, stomatal accessibility, and transport within plant tissues. Consequently, the spatial precision of droplet deposition does not necessarily translate into an equally localized physiological response [62,75]. Printed sensors and bioelectronic interfaces, in turn, generally require continuous and uniform deposited layers with excellent surface adhesion. Taken together, the droplet–plant interfacial processes provide a mechanistic basis for the following applications: growth monitoring, localized delivery, printed sensing and bioelectronics, functional biofabrication, and intelligent agricultural systems.

### 3.1. Inkjet Printing for Plant Growth and Development Monitoring

The first functional interface between inkjet printing and plant systems extends conventional text-and-image output into an in situ phenotyping tool. Unlike high-throughput platforms that rely on whole-plant imaging [76], inkjet printing deposits high-resolution fiducial markers directly on living organs in a non-contact, low-damage manner, avoiding the low precision, labor, and tissue injury of hand-drawing, adhesive tags, or needle marking [6,7]. Combined with time-lapse imaging and computer vision, these markers convert local surface deformation into quantifiable metrics of growth dynamics and stress response [77].

**Figure 3:**
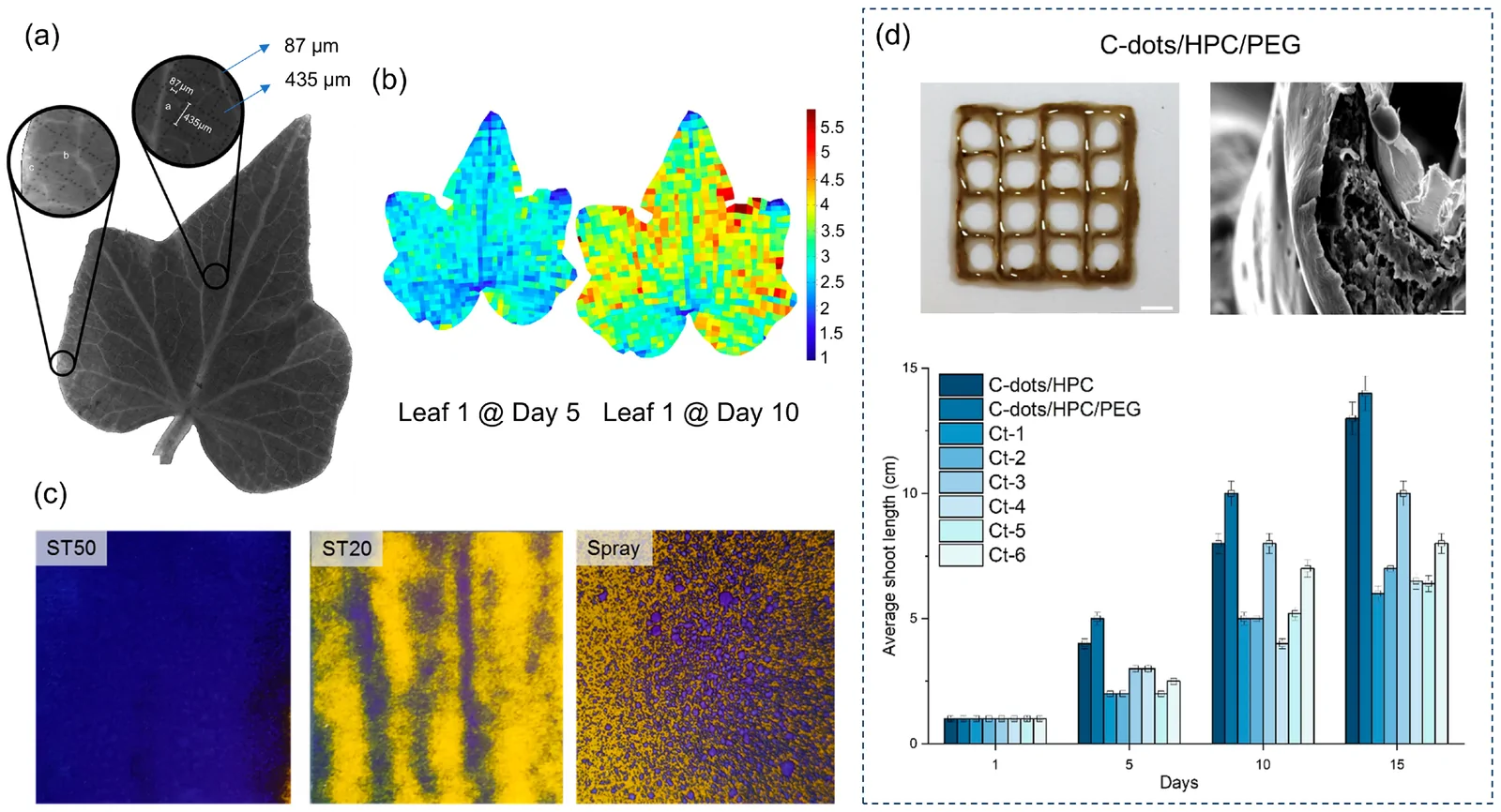
Inkjet and related printing for plant growth monitoring and precision delivery. (**a**) High-resolution inkjet-printed grid landmarks on a young ivy leaf (~700 cells; insets show dot and line spacing). Reproduced with permission from reference [6]. Copyright 2011 Springer Nature. (**b**) Expansion maps of an ivy leaf (Leaf 1) at Day 5 and Day 10 (base “cold spot”, sinus “hot spots”). Reproduced with permission from reference [6]. Copyright 2011 Springer Nature. (**c**) Coverage comparison of inkjet (ST50, ST20) vs. standard spray on hydrosensitive paper at 10 cm, showing ST50 outperforming the standard nozzle. Reproduced with permission from reference [65]. Copyright 2023 MOLECULAR DIVERSITY PRESERVATION INTERNATIONAL. (**d**) Coaxially printed carbon-dot-loaded core–shell meshes and their effects on wheat seedling growth. Top: Photograph (left; 5 mm) and SEM cross-section (right; 100 μm) of the 3D-printed mesh. Bottom: Average shoot length of wheat seedlings over 15 days. Reproduced with permission from reference [84]. Copyright 2023 American Chemical Society.

Its most direct use is printing micrometer-scale dot arrays or grids on fragile tissues such as young leaves, with reported schemes spanning custom to commercial equipment. On young ivy leaves, a grid of 40 μm dots at ~91 μm spacing divides a single leaf into ~700 cells (Figure 3a) [6]; a commercial printer can mark Arabidopsis inflorescence stems and rice seedlings with 0.1–0.2 mm dot arrays in seconds per plant [7], and a similar grid applied to sweet-pea dorsal petals provides a spatial reference for resolving the role of CYCLOIDEA in local petal growth [78]. This on-demand patterning on living surfaces also lays the groundwork for printing functional devices, such as plant electronic tattoos, directly onto leaves [79,80].

Once marked, grid-point displacements are resolved into local growth information through time-lapse imaging and computer vision. This has been used to track differential growth at petal margins and laminae and to explain the emergence of negative Gaussian curvature [78], to locate an expansion “cold spot” at the ivy leaf base and a “hot spot” at the sinus while quantifying the effect of shading on expansion (Figure 3b) [6], and to reveal near-coordinated but slightly offset elongation rhythms along Arabidopsis stem segments, in contrast to the linear elongation of rice leaves within the sheath [7].

These displacements are then converted into local strain tensors to give biologically meaningful growth fields, which are continuous fields characterized by rate, direction, and anisotropy [81]. Such analysis is widely used to quantify expansion rate, direction, and anisotropy across leaf or stem surfaces [6,7], and an analogous approach in soft-matter research reproduces lily-margin-like non-Euclidean deformation via gradients in crosslink density [82]. The resulting parameters, such as expansion rate and peak local strain, are comparable across samples and well suited to large-scale screening of growth-related genes [7]. Inkjet markers can also track stress: when the temperature was raised from 10 to 30 °C, they picked up the change in growth almost immediately [7]. Extending this capability from passive marking to active sensing devices allows inkjet printing to report strain, temperature, and even volatile signals directly, as discussed in Section 3.3.

Challenges remain in ink biocompatibility, long-term marker stability, and the difficulty of resolving three-dimensional leaf curvature from two-dimensional images. Even so, when integrated with sensing materials and imaging algorithms, inkjet printing is becoming more than a marking tool, offering a route to high-resolution growth analysis and larger-scale phenotyping.

### 3.2. Precision Delivery and Localized Manipulation

The non-contact deposition, picoliter-scale volume control, and spatial addressability of inkjet printing make it well suited to precision delivery in plant systems. Its value lies in delivering chemical, biological, or genetic payloads to target regions at predefined positions and doses, advancing conventional coarse, whole-plant treatment toward localized, programmable intervention [65].

For pesticide delivery, inkjet can reduce drift and total usage of conventional spraying, enabling microdose, targeted application. Inkjet-based delivery has been shown to be compatible with a range of herbicides, fungicides, and insecticides, achieving higher per-area operating efficiency than standard nozzles under laboratory conditions (Figure 3c) [65]. Spatially referenced crop data from autonomous lidar and hyperspectral sensing [66] could help guide future localized inkjet delivery, although this integration remains to be demonstrated.

For nutrient and hormone delivery, mature inkjet examples are still lacking, but extrusion-based 3D printing, such as coaxial direct ink writing (cDIW), offers instructive neighboring references. Core–shell mesh carriers can encapsulate multi-component fertilizers at room temperature and release them slowly, reaching about 95% release over eight weeks and boosting wheat-sprout growth roughly 1.5-fold [83]. Similar core–shell structures loaded with carbon dots increase wheat seedling growth, with shoot lengths approximately 2.5 times those of untreated controls after 15 days (Figure 3d) [84]. DIW-printed poly(vinyl alcohol)–carbomer porous hydrogels promote root development through tunable pore structure, increasing tomato root-tip number by 86.7% and total root length by 140% [85]. Together, these examples show that the “programmable structure plus controlled-release formulation” approach is shifting from delivering a single compound toward composite formulations with long-term release. These principles may also apply to inkjet, which builds up material layer by layer and deposits it on demand, although this has yet to be demonstrated for inkjet delivery of nutrients and hormones.

For biological and genetic delivery, inkjet systems can already be used for in situ inoculation of plant-growth-promoting microorganisms, with beneficial fungi and bacteria such as Trichoderma and Bacillus remaining viable after printing [65]. RNAi, CRISPR components, and nanoparticle carriers could eventually support a “print–deliver–regulate” workflow. Open issues include the effect of ink formulation on active components, transmembrane transport efficiency from leaf surface to target cells, the stability and safety of CRISPR/dsRNA under field conditions, and scale-up and regulatory requirements. Current inkjet delivery systems are therefore better suited to localized microdosing than to bulk liquid application.

### 3.3. Printed Plant Sensors and Bioelectronics

Inkjet printing provides a low-cost, maskless additive route to plant bioelectronics. Unlike conventional micro/nanofabrication, which relies on rigid substrates, vacuum processing, and photolithography, it patterns conductive structures directly onto flexible substrates and even living plant surfaces at ambient temperature and pressure [13,39]. A range of functional inks is available for these devices: metal nanoparticles such as silver and gold work as electrodes, carbon materials such as graphene and carbon nanotubes as interconnects, and conductive polymers such as PEDOT:PSS as sensing layers [13]. This variety makes inkjet a practical route towards plant-wearable devices [86].

**Figure 4:**
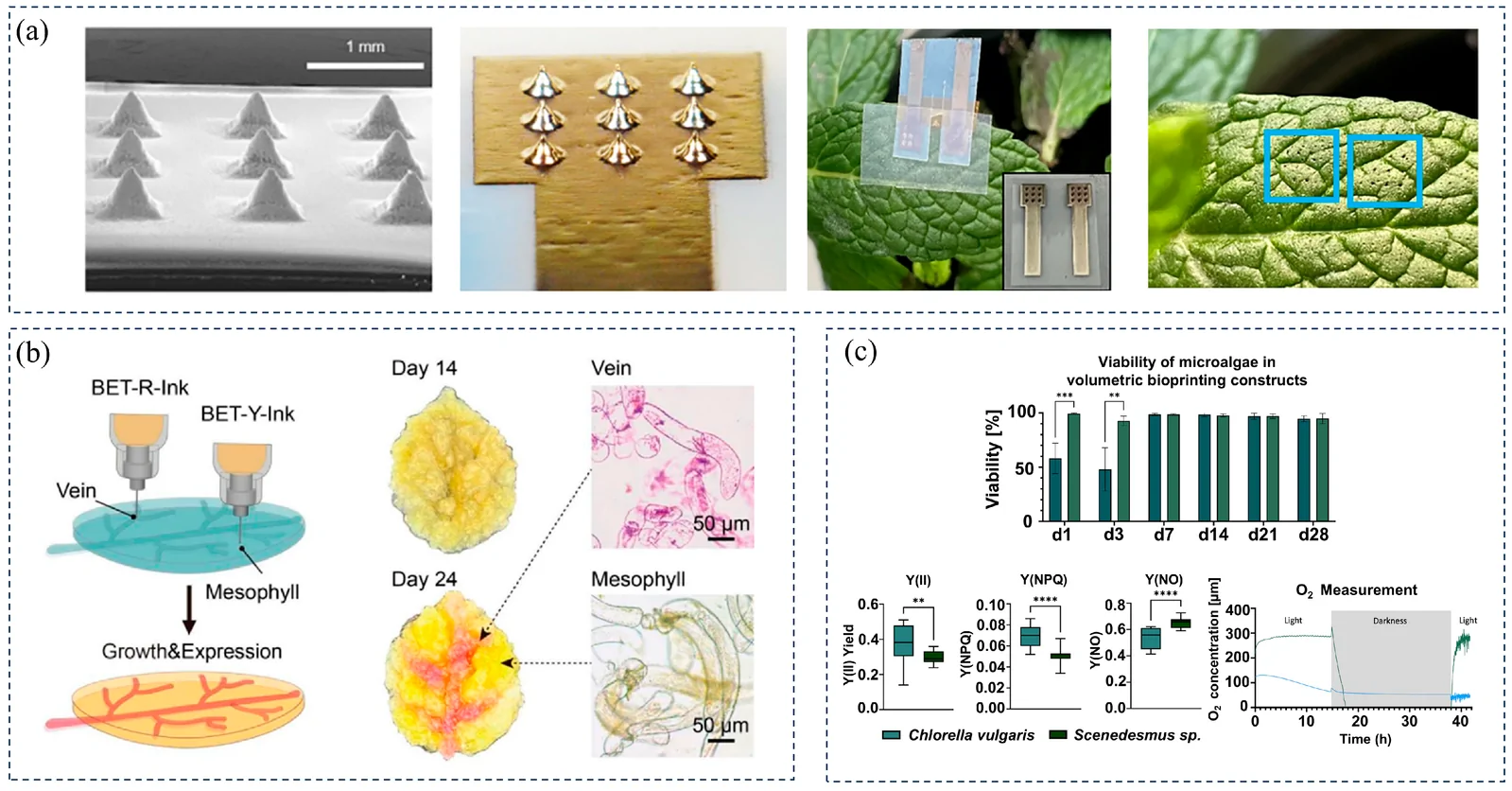
Inkjet and related printing for plant sensing, bioelectronics, and green bioprinting. (**a**) Inkjet-printed conductive microneedle arrays for leaf impedance measurements. From left to right: representative SEM image, array used for measurements, measurement setup on a mint leaf, and leaf penetration marks after measurement.Adapted with permission from reference [87]. Copyright 2024 Springer Nature. (**b**) Bioprinting of engineered tobacco BY-2 living materials with spatially patterned betalain pigmentation. Adapted with permission from reference [100]. Copyright 2024 American Chemical Society. (**c**) Viability and photosynthetic oxygen production of volumetrically printed microalgae. (Left): cell viability over 28 days (>94%); (middle): photochemical/non-photochemical quantum yields [Y(II), Y(NPQ), Y(NO)]; (right): dissolved-O_2_ during cultivation (Asterisks indicate statistically significant differences between the groups connected by brackets: ** *p* < 0.01, *** *p* < 0.001, and **** *p* < 0.0001). Adapted with permission from reference [101]. Copyright 2026 Elsevier.

Minimally invasive microneedle sensors are representative. Inkjet printing enables low-cost fabrication of three-dimensional conductive microneedles, whose drop-on-demand vertical growth, array formation, and leaf attachment are illustrated in Figure 4a [9,87]. Aerosol-jet-printed silver-nanoparticle microneedle arrays combine mechanical strength with geometric reproducibility and can penetrate the cuticle and epidermis to contact apoplastic fluid, phloem, or xylem [39]. Beyond serving directly as conductive in situ sensing interfaces, microneedles can also act as minimally invasive sampling ports: PEG-crosslinked hydrogel microneedle patches, inserted into leaves and combined with centrifugation, rapidly extract tomato leaf sap for on-site hydrogen peroxide detection with a portable photoelectric colorimeter [88]. Microneedle platforms can thus both retrieve internal plant fluids with low damage and couple with electrochemical sensing to detect markers such as pH and hydrogen peroxide, providing internal information on stress and metabolic responses [88,89].

Inkjet printing can also be used to make pesticide-residue detection devices. For example, printed acetylcholinesterase membranes combined with quantum-dot microarrays form test cards that detect organophosphate pesticides by a visible color change, with detection limits below national standards [90,91]. Beyond sensing-material design, practical plant-monitoring devices require integration of electrical interconnects, readout circuitry, power provision, and data communication. Inkjet-printed conductive tracks and antennas provide building blocks for RFID and sensing electronics [92]. In a plant-specific example, Baraniecki et al. [39] fabricated aerosol-jet-printed microneedles on inkjet-printed tracks and mounted them alongside environmental sensors on a wooden clip. Anisotropic conductive tape connected the sensing patches to flexible Kapton NFC tags containing SIC4340 sensing chips, enabling electrical measurements and battery-free wireless readout through a smartphone application. This approach combines printed sensors, conventional electronics, and mechanical supports in an integrated plant-monitoring device. However, readout requires a nearby smartphone, rather than providing autonomous, continuous field monitoring.

A further direction is the wearable “plant electronic skin” (e-skin), which conforms to leaves, stems, and fruit to track parameters such as growth strain, surface temperature, humidity, and volatile organic compounds [93]. Printing relative-humidity sensors directly on leaves has shown that the plant itself can serve as a functional substrate [79]; building on this, a leaf-conformal Janus e-skin exhibits adhesion to plant leaves and monitors growth strain, temperature, and ethylene release in fully encapsulated and semi-open modes, achieving ~96.8% signal-classification accuracy with a CNN [94]; liquid–metal plant electronic tattoos remain conformal during dehydration-induced leaf shrinkage [79]; and inkjet-printed low-temperature gel implants achieve months-long monitoring of ionic activity in stem tissue [95]. Evidence of adhesion and environmental tolerance has also been reported in related plant-wearable systems. Yang et al. [80] demonstrated leaf adhesion under wilting, water-flow, and immersion conditions, and separately used their all-organic e-skin for on-plant monitoring under combined heat and drought stress. Stretchable polymer sensors have also shown resilience to humidity [96]. Despite these advances, practical deployment still faces several challenges. High-temperature sintering may damage living tissue, motivating low-temperature processing and biocompatible inks. Adhesion, delamination, and signal drift during plant growth remain concerns, while long-term microneedle signal fidelity, wound responses, and NFC read range require further assessment. Durability under fluctuating temperature and humidity, rain, UV exposure, and wind-induced leaf motion also needs validation for the specific device and plant interface. Printed microneedles and e-skins therefore offer promising routes toward deployable plant monitoring, although long-term field performance remains to be established.

### 3.4. Green Bioprinting: Toward Engineered Living Materials

When the printing target extends from sensors and small molecules to living plant cells, the technical goal shifts from surface fabrication to the construction of living materials. This is the core of green bioprinting: using inkjet or extrusion printing to directly organize plant cells, algae, or related matrices into constructs capable of growth, metabolism, or environmental response [97]. Within this broad notion of green bioprinting, inkjet mainly handles fine manipulation at the single-cell level and nanoscale interfaces [98], whereas centimeter-scale living structures are built mostly by extrusion or volumetric printing.

Maintaining cell viability is the primary challenge. The plant cell wall confers some mechanical tolerance, but the large vacuole renders cells shear-sensitive, and non-uniform cell-cluster size constrains nozzle choice and resolution [99]. The corresponding strategy is to match the cell source to the nozzle: suspension culture yields a near-single-cell state, and choosing a nozzle that accommodates > 85% of the cell clusters (i.e., a critical fraction > 85%) enables centimeter-scale fine structures to be printed at >80% viability [99]; protoplasts, though somewhat lower in short-term viability, are more amenable to cell fusion and genetic modification once the wall is removed [97]. Inkjet is especially valuable for single-cell–nanostructure manipulation: a bioink of Chlamydomonas reinhardtii with 10 vol% dimethyl sulfoxide exploits droplet dynamics to stably insert vertically aligned nanowires into algal cells, opening new routes for photosynthetic electron harvesting and subcellular operation [98]. Extrusion printing, in turn, demonstrates an integrated “print–culture–transform” strategy: a tobacco BY-2 suspension-cell granular hydrogel scaffold, after Agrobacterium transformation, both supports long-term cell growth within the 3D scaffold and introduces a betalain biosynthesis pathway to form tunable pigment patterns (Figure 4b) [100].

Extending the target to photosynthetic algae gives green bioprinting an autotrophic character: microalgae such as Chlorella and Scenedesmus embedded in hydrogel scaffolds continue to produce oxygen and fix carbon after printing. Volumetric bioprinting with a poly(ethylene glycol) diacrylate–cellulose nanofibril resin can build complex 3D structures with high cell density (>94% viability) that maintain photosynthetic efficiency over the long term, with oxygen output already used to assess environmental and space-application potential (Figure 4c) [101]. Bioprinting can therefore go beyond cell immobilization toward engineered living materials with metabolic function.

In food manufacturing, this concept points toward renewable food: edible inks loaded with plant callus can form tissue-like structures after printing and culture. Carrot callus mixed with alginate ink sustains cell growth over 35 days, forming cell clusters with tunable texture (with the potential to mimic real-food texture and nutrition) [102].

The longer-term goal is on-demand manufacturing of plant-based living materials. Through biomimetic structuring and oriented fiber alignment, freeze direct-writing of aligned cellulose-nanocrystal inks can construct customized wood-like aerogel structures [103]; living hydrogels embedding photosynthetic algae have been used for carbon-dioxide capture, wastewater treatment, and oxygen supply [104], potentially serving as functional modules in building facades or closed-loop life-support systems.

Green bioprinting nonetheless remains far from large-scale application: the effects of extrusion pressure and needle diameter on viability [105], sterility control, cell-wall and totipotency regulation, morphological stability during long-term culture, and food-grade material safety review [106] are all bottlenecks to overcome, while light penetration at high cell density and uniform illumination of large structures [101] will directly determine practical utility. The field remains at the stage of laboratory-scale integrated validation. In sum, plant biomanufacturing is moving from simply positioning cells toward building living materials. Inkjet and related printing technologies could help create plant and algal structures that grow on their own, regenerate, and can be tailored to specific functions. This would give cellular agriculture, sustainable manufacturing, and engineered living materials a new set of tools.

### 3.5. Inkjet-Integrated Intelligent Agricultural Systems

**Figure 5:**
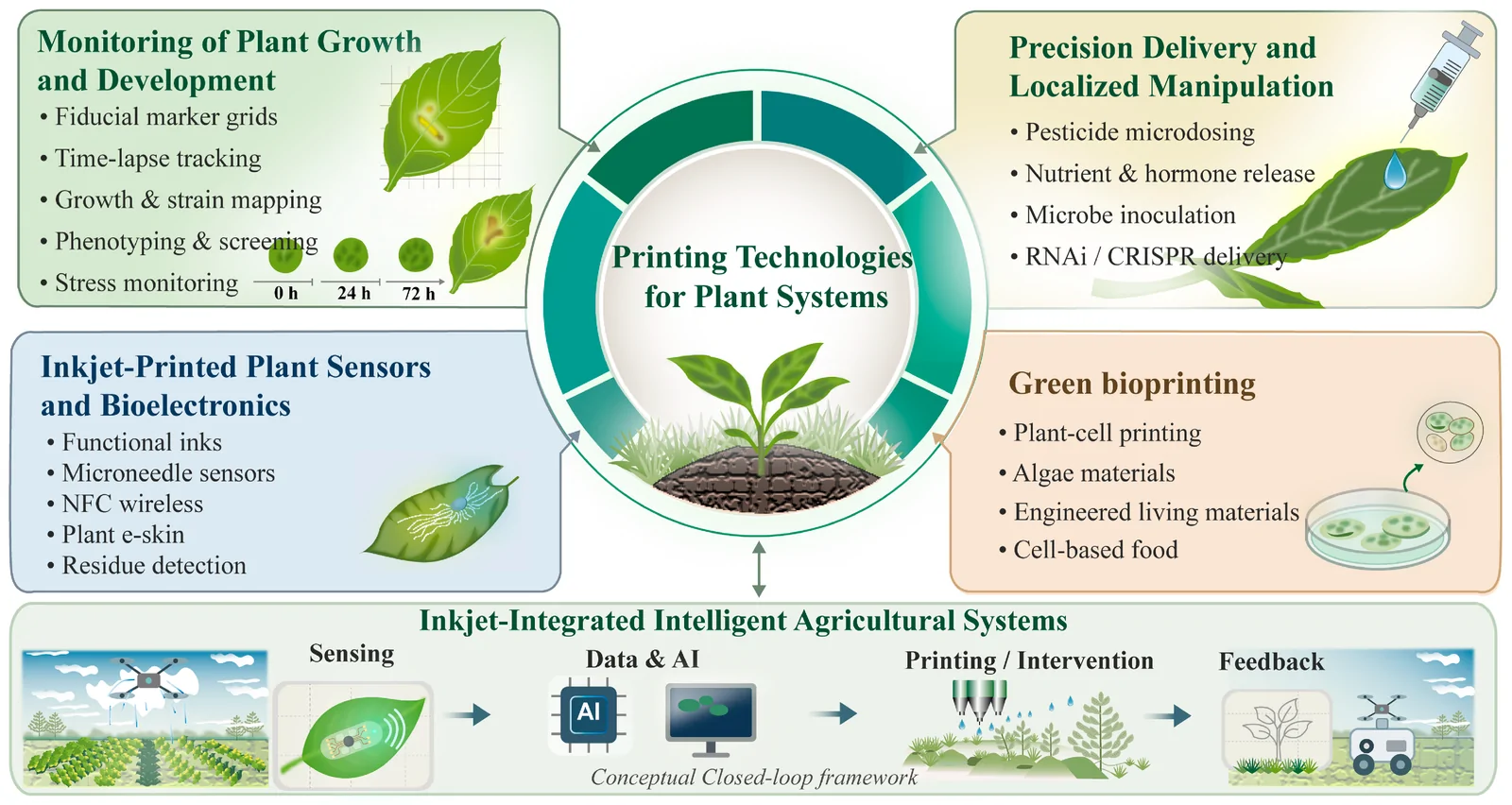
Functional interfaces between printing technologies and plant systems.

Inkjet printing may eventually provide a precise delivery component for intelligent plant-management systems. In a closed-loop framework, sensors would monitor plant status, algorithms would identify the location and dose of an intervention, and a printing device would deposit the selected material. However, an integrated system combining these functions has not yet been demonstrated. Figure 5 summarizes the application areas reviewed in this section and illustrates a conceptual closed-loop framework for their future integration.

Current applications are limited mainly to on-demand delivery and print-process control. Plant sensing still relies largely on remote-sensing platforms and wearable devices, including liquid–metal electronic tattoos [79] and microfluidic stomatal sensors [5]. The Ladybird platform combines crop sensing and autonomous navigation with a robotic arm and a nozzle-based unit for targeted spraying [66], although this configuration uses a spray nozzle rather than an inkjet printhead. Inkjet-based delivery has been demonstrated for selected pesticides and beneficial microorganisms under controlled conditions [65]. Algorithm-assisted waveform optimization has also been used to improve droplet stability and print quality [107]. Printed cryogel implants and leaf-surface electronic skins may provide sensing components for future feedback systems [94,95], but their integration with inkjet-based delivery remains to be validated.

This section therefore describes a possible direction rather than an established agricultural system. Closed-loop operation will require the reliable integration of sensing, target recognition, positioning, and deposition, together with improvements in throughput and field robustness. Inkjet delivery may eventually be extended to nutrients, growth regulators, RNA-based agents, and other advanced payloads, but these applications still require direct validation in living plants.

## 4. Cross-Disciplinary Challenges

Inkjet printing has clear potential across plant monitoring, delivery, electronics, and integrated smart systems. Moving from laboratory proof-of-concept to field deployment, however, requires addressing the coupled problems that arise where plant biology, materials science, precision manufacturing, and data intelligence meet. This section examines the key challenges from four perspectives: the plant system, the ink–plant interface, multiscale integration, and AI-based control. These four challenges follow one thread: the specificity of the living interface constrains how inks can be designed; these constraints are further magnified at the field scale, and closing the resulting control loop ultimately depends on data and AI.

### 4.1. Plant-System Specificity

The heterogeneous wettability, roughness, and curvature of living plant surfaces make droplet deposition strongly dependent on organ type, surface position, and developmental stage. Unlike static engineering substrates, these interfacial properties also change continuously during plant growth, making a single set of printing parameters difficult to transfer across species, organs, and growth stages [61].

In time, the leaf is an active interface that continuously exchanges water and solutes: transpiration and bidirectional cuticular water flux change the local humidity and solute concentration near the droplet, diluting active components, altering drying and rheological behavior, and affecting permeation through the cuticle or stomata [62,63]. Growth continues after printing, and organ expansion makes existing markers, sensors, or delivery sites drift spatially and lose registration. For attached devices, leaf expansion keeps introducing strain at the device–tissue interface; once the modulus is mismatched, this strain concentrates at the device–tissue bond and readily leads to adhesion failure or signal drift, which makes low-modulus, stretchable designs that deform with growth particularly important [96]. The cell wall is also remodeled during development, with stiffness and chemical composition varying with developmental stage and stress [108,109], which works against the long-term stable readout that devices require. In addition, mechanical contact, microneedle penetration, or certain ink constituents can trigger wound signaling and jasmonate-mediated defense, engaging the growth–defense trade-off [110], while long-term in vivo implantation may further induce fibrosis-like encapsulation [95].

The target of inkjet printing is therefore a heterogeneous, dynamic, and actively responding living interface. Instead of fixed parameters calibrated once, a more workable approach is adaptive printing, in which plant growth models and real-time physiological status are placed in a parameter feedback loop so that droplet volume, landing position, and curing conditions adjust to leaf topography, hydration state, and developmental stage.

### 4.2. Ink–Plant Interface

Inks for plant use must reconcile functional output with biocompatibility, and their design has to balance materials chemistry, interfacial physics, and plant physiology. The first difficulty is the trade-off between toxicity and performance. Functional nanomaterials may pose composition- and dose-dependent risks to plants. For silver-based nanomaterials, including effects associated with released Ag^+^, reported responses include tissue uptake, oxidative stress, and interference with photosynthesis and water transport [111]. Natural matrices such as alginate and cellulose show good compatibility but insufficient performance. This tension among rheology, structure, and biological function has been described as an intrinsic trade-off of bio-inks [64]. Microalgae-based bio-inks show the same logic: synthetic polymers such as PEGDA improve structural integrity and shape fidelity, but potentially at the cost of cytocompatibility and permeability [112].

The second difficulty is that permeation and toxicity are linked: highly permeating carriers or surfactants help components cross biological barriers, but they also raise local toxicity, because the same weakening of the cuticular barrier that aids uptake also exposes underlying cells to the active agent [62]. At the same time, deposits must stay attached to a soft, moist, growing surface. Liquid–metal tattoos, for example, can wrinkle as they dehydrate yet remain conformal [79], whereas uncontrolled ink spreading or migration of the surface water film causes lateral droplet diffusion and weakens spatial precision [61]. Beyond ink composition, the printing process itself may also affect biological systems. In a LEMS configuration, LIFT-based laser bioprinting altered membrane permeability in viable yeast cells and caused a modest reduction in cell viability [113]. Such permeabilization may facilitate the uptake of molecules or genetic material, although its duration and practical value remain to be established. Whether similar effects occur in plant cells remains unclear, particularly because of their cell walls, large vacuoles, and osmotic sensitivity.

Plant inks therefore cannot be designed from material performance alone. They call for interface-cooperative design, in which viscosity, surface tension, toxicity, permeability, adhesion, and degradation behavior are optimized together as a coupled system for a specific species, organ, developmental stage, and target function. Accordingly, compatibility assessment for plant-oriented inks should extend beyond printability and acute toxicity to physiological indicators such as photosynthetic performance, stomatal behavior, transpiration, oxidative stress, membrane integrity, and longer-term growth responses.

### 4.3. Multiscale System Integration

A further tension lies between microscale manufacturing precision and macroscale agricultural deployment. In the laboratory, inkjet and EHD printing can achieve micro/nanoscale patterning for microelectrode arrays, cellular micropatterning, and related uses [98,114], but this precision depends heavily on strict control of the environment and process.

Field conditions add uncertainty. The controlled temperature and humidity, vibration isolation, and fixed, planar substrate that hold in the laboratory no longer apply: nozzles and piezoelectric elements are sensitive to dust, vibration, and temperature change, and hectare-scale operation requires extending picoliter-level droplet control to large areas, usually with robots, gantry systems, or unmanned aerial vehicles [66]. Robotic-arm positioning, flight stability, and jetting consistency during motion then constrain one another. In EHD printing, for example, vibration and substrate offset cause fluctuations in nozzle spacing and electric field, which can be brought to a usable level only through real-time compensation such as a self-stabilizing electric field [67]. Lab-to-field translation thus depends less on the print head than on whether addressing, uniformity, robustness, and throughput can be maintained together under wind, light, rain, and dust, which in turn requires modular platforms and real-time compensation.

### 4.4. Data and AI

A closed-loop system must also coordinate data, algorithms, and actuation. Even when microneedle pH sensors capture rapid changes in the laboratory [40], the accumulated latency of signal processing, transmission, inference, and nozzle response still makes actuation lag behind the plant’s true physiological state. On the data side, canopy images, in situ signals, and soil data differ in scale, dimensionality, and noise, and a fusion framework built for plant systems is still lacking [115]. A deeper bottleneck is robustness: recognition models are largely trained under controlled conditions and tend to fail under interspecific variation, developmental change, and combined stresses, while black-box models can hardly explain how intervention-induced defense responses feed back to change their own predictions [116].

AI could therefore extend beyond downstream analysis and be integrated with models of plant state and printing actuators. In the longer term, such integration may support plant digital twins capable of estimating plant status and adjusting interventions adaptively [116]. Printing technologies and living plants form a closely coupled system, and field-scale implementation requires cross-disciplinary design of printing parameters, ink materials, hardware platforms, and control algorithms.

## 5. Future Perspectives: From Printing Technologies to Integrated Plant Systems

Growth monitoring, precision delivery, plant electronics, and biofabrication may together enable more precise and responsive ways of working with living plants. In this context, the value of inkjet and related printing technologies extends beyond material deposition to their potential for precise, low-damage intervention in plant processes. The contribution of this review is to connect printing capabilities with ink formulation, plant-interface behavior, and application requirements, rather than considering deposition precision alone. This framework helps distinguish demonstrated plant applications from capabilities inferred from other systems and identifies where plant-specific validation is still needed.

### 5.1. Printing-Enabled Localized Intervention

A near-term opportunity lies in extending printing from one-time localized deposition toward more adaptive intervention. Thermal and piezoelectric inkjet printing are particularly relevant because their drop-on-demand operation enables precise control of deposition position and dose. Such capabilities could support localized delivery of nutrients, pesticides, hormones, microorganisms, or other active materials [65]. However, moving from controlled deposition to predictable biological regulation requires direct validation of dose–response relationships, transport across plant surfaces, and long-term physiological effects. Thus, the immediate priority is not to make plants “programmable”, but to establish reproducible links between printing parameters, delivered dose, and plant response. A practical next step is to evaluate formulation–process–response relationships in the same plant system. Additive screening should assess jetting and deposition alongside payload activity, tissue compatibility, and subsequent growth, so that improved printability can be linked to a biological benefit.

### 5.2. Integration of Printing with Sensing and Feedback

A further opportunity is to integrate printing with plant sensing and feedback control. Plant-wearable and microneedle sensors can provide information on growth, water status, ionic activity, and stress, while algorithms may determine when and where subsequent deposition is required [39,94,116]. Such integration would enable printing systems to move beyond predefined deposition toward more adaptive interventions, where delivery strategies are adjusted according to plant status. Realizing this capability will require stable long-term sensing, accurate interpretation of plant physiological states, and direct validation of the relationship between sensor outputs, printing operations, and plant responses.

### 5.3. Living Agricultural Interfaces

In the longer term, printing may contribute to integrated plant interfaces combining flexible electronics, responsive materials, sensing, and localized delivery. The four printing approaches considered here offer complementary capabilities: thermal and piezoelectric inkjets provide mature low-volume drop-on-demand deposition; AJP is attractive for conformal, high-resolution conductive features; and EHD printing offers finer patterning and broader material processing but requires more demanding electrical and process control. Their integration with living plants will ultimately depend on maintaining adhesion, biocompatibility, signal stability, positioning accuracy, and sufficient throughput during plant growth and under field conditions.

## 6. Conclusions

Overall, the printing technologies reviewed here occupy complementary rather than interchangeable roles in plant systems. Thermal and piezoelectric inkjet printing provide mature drop-on-demand deposition, AJP offers conformal high-resolution fabrication particularly suited to plant bioelectronics, and EHD printing extends feature resolution and material range but remains constrained by high electric fields and limited plant validation. Across these technologies, practical performance cannot be assessed from nominal resolution or jetting frequency alone. It also depends on the feature size actually achieved, throughput at the organ scale, the risk of printing-induced biological damage, the physiological compatibility of the inks with plant tissues, and the interfacial behavior of droplets on plant surfaces.

Current experimental evidence supports several concrete applications, including surface marking and growth monitoring on living plant organs, fabrication of plant-interfacing sensors and conductive structures, and localized delivery of selected chemical or biological agents. However, the level of validation differs among applications, and direct evidence remains limited for printed delivery of nutrients, hormones, genetic materials, and other advanced payloads. These applications therefore require further plant-specific validation before broader claims of physiological regulation can be made.

More integrated concepts, including programmable plants, autonomous feedback systems, plant digital twins, and intelligent agricultural interfaces, remain prospective rather than established capabilities. Their realization will depend on reliable sensing, reproducible plant responses, stable living interfaces, sufficiently rapid actuation, and field-scale robustness. Future work should therefore prioritize direct validation of the relationships between printing parameters, delivered dose, and plant responses, while more integrated systems remain longer-term research directions.

## Figures and Tables

**Table 1:** Comparison of printing technologies for plant systems.

Ref.	Printing Technique	Droplet Jetting Mode	Ink Type	Ink Viscosity	Jetting Precision (Droplet Size/Resolution)	Jetting Frequency	Biocompatibility	Scalability & Deployability	Applications
[17]	Thermal inkjet	Drop on demand	CHO cell suspension	Low-viscosity aqueous	~130 pL	-	Low thermal damage; post-printing viability > 90%	Mature, low-cost, scalable	Live-cell printing; biofabrication
[18]	Thermal inkjet	Drop on demand	Bio-ink (glucose oxidase, GOD) Electronic ink (PEDOT:PSS conductive polymer)	Low-viscosity aqueous (glycerol as viscosity modifier)	10–12 pL per drop; nominal 1200 dpi, measured 221 × 221 dpi	-	No loss of enzyme activity before/after printing (>90% retained)	Low-cost; toward scalable biosensors	Amperometric glucose biosensor
[19]	Thermal inkjet	Drop on demand	Bio-ink (C2C12 mouse myoblast suspension)	Low-viscosity aqueous (PBS)	85 μm (300 dpi); ~one cell per droplet	-	Cell viability 91.2 ± 2.6%	Low-cost; no complex processing	Muscle-actuated Bio-MEMS biosensors; tissue engineering
[20]	Piezoelectric inkjet	Drop on demand	Neural cell suspension (Schwann/NG108-15)	Low-viscosity aqueous	pL-scale (nozzle diameter of 60 μm)	-	Non-thermal; cell viability 82–96%	Mature, moderate cost, scalable	Neural cell printing; tissue engineering
[21]	Piezoelectric inkjet	Drop on demand	ZnO suspension PEDOT-based solution	200 mPa·s	~21 pL	-	Non-thermal; biocompatibility ink-dependent	Moderate maturity, moderate cost	Advanced functional-material deposition
[22]	Piezoelectric jetting (single-piezoelectric-stack jet dispenser)	Drop on demand	Liquid wax Honey UV polymer Epoxy resin Silver paste Ceramic slurry	21–665,320 mPa·s (spanning > 4 orders of magnitude)	Minimum droplet volume ~14.4 nL	Up to 500 Hz (measured 50–333 Hz)	Non-contact; temperature-tunable heating block; prints soft substrates; no live cells printed	Wide-viscosity adaptability; simplified single-stack structure; good engineering maturity	Lubricants; food; adhesives; electronic packaging; conductive silver; ceramics; 3D printing
[23]	Piezoelectric inkjet (squeeze-type)	Drop on demand	Nano-silver conductive ink (Jet-600C)	6 mPa·s	Droplet diameter ~29.2134 μm (~half the nozzle diameter; nozzle diameter of 60 μm)	1 kHz (driving frequency)	Non-thermal (piezoelectric); no living tissue; conductive-pattern oriented	Single nozzle; lab-scale waveform-optimization study; not yet scaled	Conductive patterns; printed & flexible electronics (conformal antennas, wearables, biosensors)
[24]	Piezoelectric inkjet	Drop on demand	Enalapril maleate (EM) PEG400 drug ink (1.25% aqueous, 10% methanol-based)	7–17 mPa·s	native resolution 50 dpi (nozzle diameter of 30 μm)	500–1000 Hz	Non-thermal (piezoelectric); printed on edible films, non-living printed	Continuous in-line manufacturing; high industrial readiness	Orodispersible-film personalized dosing; pediatric dosing; fixed-dose combinations
[25]	Aerosol jet printing	Direct writing	AgCl conductive ink (XA-3773) carbon ink (EXP 2652-28) MWCNT ink GOD (glucose oxidase) ink NOA 81 UV-curable polymer	AgCl diluted to ~19.5 mPa·s (23 °C); carbon ink ~250 mPa·s	Line width/resolution 50–100 μm	NA	Non-contact, room temperature; mediator/enzyme directly printed with retained activity; biofluid detection (no live cells)	Relatively high equipment cost	Electrochemical glucose biosensor
[26]	Aerosol jet printing	Direct writing	Silver (Ag) nanoparticle conductive ink	AJP wide viscosity; no single value	Aerosol droplets 1–1.5 μm; deposited line width ~7 μm	NA	Non-contact, room temperature; electronics-oriented	Complex equipment; carrier gas 5–20 sccm	Conformal electronics; high-density circuits, resistors, conformal electrodes
[27]	Electrohydrodynamic printing	Direct writing	High-viscosity copper paste (Cu paste: PGMEA = 100:1)	4000 mPa·s	Minimum line width ~40 μm (nozzle inner diameter of 100 μm)	NA	Requires kV-level strong field	Low-cost plastic nozzle, limited scalability (vacuum annealing)	Printed copper electrodes; thin-film transistors (TFTs); flexible electronics
[28]	Coaxial Electrohydrodynamic printing (CEHD)	Direct writing	Inner liquid: PZT sol Outer liquid: silicone oil (coaxial dual fluid)	PZT sol 160 mPa·s; silicone oil 58.56 Pa·s	Suspended PZT nanowires ~100 nm	NA	Strong field but low voltage (5 kV); MEMS-oriented	Low operating voltage (5 kV)	MEMS; nano force/energy sensors; flexible piezoelectric devices
[29]	Electrohydrodynamic printing	Direct writing	20%/25%/30% PVA/DMSO hydrogel ink	Up to ~10,000 mPa·s	Line width ~64.15 ± 4.57 μm	NA	Biocompatible PVA hydrogel; biomedical-oriented; ink heated to 85 °C, substrate cooled to 8 °C, 4 kV	Low-cost; easy to use	High-resolution PVA hydrogel scaffolds; biomedicine/tissue engineering
[30]	Multi-nozzle Electrohydrodynamic printing	Drop-on-demand	Triethylene glycol (TEG) ethanol silver ink quantum dots	Triethylene glycol 50 mPa·s; ethanol 1.1 mPa·s	100 dpi (nozzle diameter of 45–80 μm)	10 kHz	Strong field; electronics-oriented	Large-scale, high-density strongest scalability	Printed electronics; wearables; flexible displays

Note: Values are as reported in the original studies under differing inks and conditions, and are indicative rather than strictly comparable. Examples are representative, not exhaustive; many come from mammalian-cell, biomedical, or electronic systems and primarily serve to illustrate process capabilities and engineering constraints, so transfer to plants remains unverified. Biocompatibility, maturity, cost, and scalability are the authors’ qualitative assessments. “-”, not reported; “NA”, not applicable; first column (Ref.), reference number. DOD, drop-on-demand; AJP, aerosol jet printing; EHD, electrohydrodynamic; CEHD, coaxial EHD; MEW, melt electrowriting; GOD, glucose oxidase.

**Table 2:** Dimensionless parameters governing inkjet droplet formation and their implications for plant-oriented ink design.

Parameter	Physical Meaning	Relevance to Printing	Implications for Plant-Oriented Ink Design
*Re*	Ratio of inertial to viscous forces	Influences the flow regime and shear effects during droplet jetting	High jetting velocities may increase shear-related damage to suspended cells or protoplasts. Therefore, droplet jetting conditions should be balanced against cell viability [35,36].
*We*	Ratio of inertial to capillary forces	Influences droplet impact and spreading	Plant leaves often have hydrophobic and waxy surfaces. A high *We* may promote droplet rebound or roll-off. Therefore, the formulation should provide suitable surface tension, while the potential phytotoxicity of surfactants should also be considered [61,62].
*Oh* or (*Z* = 1/*Oh*)	Relative contribution of viscous forces to inertial and capillary forces	Defines the operating window for stable droplet formation; (*Z*) is commonly used to assess printability	Cell-containing or agrochemical inks should remain within the printability window. Higher viscosity may help protect cells. However, it may also increase the risk of nozzle clogging. Therefore, nozzle size should be balanced against cell viability [34,35,64].

## Data Availability

No new data were created or analyzed in this study. Data sharing is not applicable to this article.
